# Surgical anatomy of the lingual nerve for palate surgery: where is located and how to avoid it

**DOI:** 10.1007/s00405-022-07432-5

**Published:** 2022-06-30

**Authors:** Octavio Garaycochea, Peter Baptista, Marta Calvo-Imirizaldu, David Terrasa, Antonio Moffa, Manuele Casale, Juan Alcalde, Carlos O’Connor-Reina, Guillermo Plaza, Secundino Fernández

**Affiliations:** 1 Department of Otorhinolaryngology, Clinica Universidad de Navarra, University of Navarra, Pamplona, Spain; 2 School of Medicine, Campus Bio-Medico University, Unit of Integrated Therapies in Otolaryngology, Fondazione Policlinico Universitario Campus Bio-Medico, Rome, Italy; 3 Otorhinolaryngology Department, Hospital Quironsalud Marbella, Marbella, Spain; 4 Otorhinolaryngology Department, Hospital Universitario de Fuenlabrada, Universidad Rey Juan Carlos, Madrid, Spain; 5 Otorhinolaryngology Department, Hospital Clínic de Barcelona, c/ Paris 146-3º-2ª, 08036 Barcelona, Spain

**Keywords:** Lingual nerve, Palate surgery, Obstructive sleep apnea, Snoring, Barbed suture

## Abstract

**Purpose:**

To describe the anatomic relationship of the lingual nerve with the lateral oropharyngeal structures.

**Methods:**

An anatomic dissection of the lateral oropharyngeal wall was conducted in eight sides from four fresh-frozen cadaveric heads. Small titanium clips were placed along the lingual nerve and the most anterior and medial border of the medial pterygoid muscle. Radiological reconstructions were employed for optimal visualization; the coronal view was preferred to resemble the surgical position. The distance between the lingual nerve and the medial pterygoid muscle at its upper and lower portion was measured radiologically. The trajectory angle of the lingual nerve with respect to the pterygomandibular raphe was obtained from the intersection between the vector generated between the clips connecting the upper and lower portion of the medial pterygoid muscle with the vector generated from the lingual nerve clips.

**Results:**

The mean distance from the upper portion of the medial pterygoid muscle and superior lingual nerve clips was 10.16 ± 2.18 mm (mean ± standard deviation), and the lower area of the medial pterygoid muscle to the lingual nerve was separated 5.05 ± 1.49 mm. The trajectory angle of the lingual nerve concerning to the vector that describes the upper portion of the most anterior and medial border of the medial pterygoid muscle with its lower part was 43.73º ± 11.29.

**Conclusions:**

The lingual nerve runs lateral to the lateral oropharyngeal wall, from superiorly–inferiorly and laterally–medially, and it is closer to it at its lower third.

## Introduction

Obstructive sleep apnea (OSA) is a sleep disorder characterized by recurrent upper airway obstruction during sleep. Considered a highly prevalent disorder and a risk factor for cardiovascular disease, its first-line treatment is continuous positive airway pressure (CPAP) [1]. However, in patients without treatment adherence and in those without a good response to other conservative treatments such as mandibular advancement device (MAD), positional therapy or myofunctional therapy, surgical treatment is an option [2, 3].

The soft palate and lateral pharyngeal walls are considered one of the primary collapse sites during sleep in patients with OSA. Moreover, soft palate vibration has also been related to snoring in patients without a partial or complete upper airway obstruction. Since Quesada and Perelló in 1979 [4] and Fujita in 1981 [5], different surgical techniques of palatopharyngoplasty have been described. In 2008, Hur introduced the sling snoreplasty with a permanent thread, a technique based on retention sutures along the soft palate to make it more rigid and pull it forward and upward [6]. Later, Montovani would improve this technique by introducing barbed sutures (BS) and anchoring the sutures to bony structures (pterygoid hamulus and posterior nasal spine) [7, 8]. Following the same line, Vinici in 2015 described the Barbed reposition pharyngoplasty (BRP), adding an anterior and lateral mobilization of the palatopharyngeal muscle [9]. Many surgeons have discovered the advantages and unique properties of the BS and allowed the popular surgical pharyngoplasty techniques to be updated and improved [10].

**Fig. 1:**
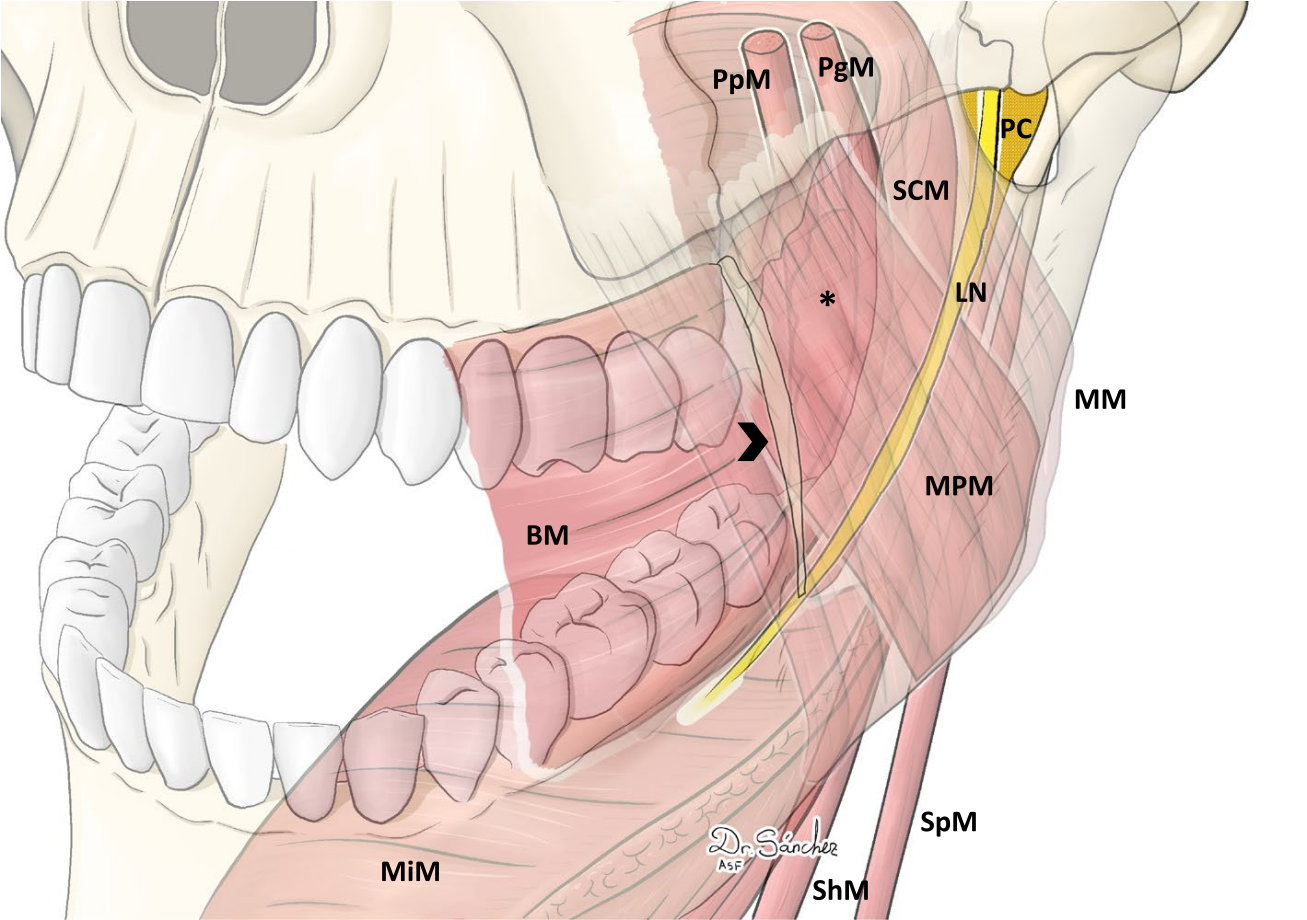
Oropharynx antero-lateral view. The relation between the PR and the LN is illustrated. Black arrow head: pterygomandibular raphe, *LN* lingual nerve, *SCM* superior constrictor muscle, *BM* buccinator muscle, *MPM* medial pterygoid muscle, *MiM* Mylohyoid muscle, *ShM* stylohyoid muscle, *SpM* stylopharyngeus muscle, *PC* prestyloid compartment. In a deeper plane the tonsil (black asterisk), the *PpM* palatopharyngeus muscle, and the *PgM* palatoglossus muscle are represented. The *MM* masseter muscle is located in a more superficial layer of the figure

Although the use of BS has evolved and will continue to do so, the anchoring points remain the same [10–12]. One anchoring point frequently described in the literature in the lateral wall is the pterygomandibular raphe (PR) [13]. The PR is considered a tendinous band of the buccopharyngeal fascia that courses from the apex of the hamulus of the medial pterygoid plate to the posterior limit of the retromolar trigone of the mandible [14]. The PR provides attachment along its anterior aspect for a portion of the buccinator muscle (BM), and from its posterior aspect to the superior constrictor muscle (SCM). However, anatomical studies have shown that the presence of this structure is not always constant, and that it is only identifiable in a third of adults [15]. The PR or the accentuated mucosal fold in the oral cavity where the SCM and BM converge in subjects without PR, lies medial to the prestyloid compartment (PC) [16]. At this level the PC contains exclusively fat tissue and the anterior and medial portion of the medial pterygoid muscle (MPM), on which the lingual nerve (LN) runs on its lateral side [16–18] (Fig. 1). Since the LN can be damaged during palatal surgery when using PR as a fixation point, especially at its caudal third [12], it is essential to know its orientation and its relationship with the lateral oropharyngeal walls. This study aims to analyze the anatomic relationship of the LN with the lateral oropharyngeal structures and also its orientation and distance from the PR.

## Material and methods

Eight sides from four fresh-frozen cadaveric heads (one female and three males) were used in this study. The cadaveric specimens were from the University of Navarra School of Medicine’s Anatomical Donor Program. Donors ranged in age from 50 to 90 years at the time of death. The cause of death was not identified for this study. The cadaveric procedures did not require specific approval by the University Ethics Committees.

### Anatomic examination

**Fig. 2:**
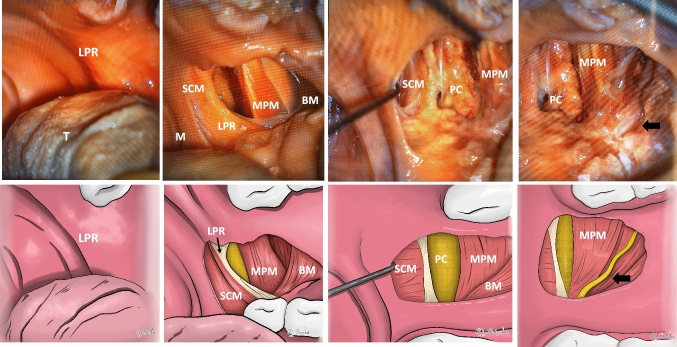
Anatomic dissection. *LPR* left pterygomandibular raphe, T tongue, *SCM* superior constrictor muscle, *M mucosa* MPM medial pterygoid muscle, *BM* buccinator muscle, *PC* prestyloid compartment. Black arrow: lingual nerve

The anatomic dissection was performed with the aid of an operating microscope (Zeiss OPMI VISU S7). A mucosal incision was made along the accentuated mucosal fold in the oral cavity at the PR level. A Rosen knife curette was used to dissect the mucosa over the muscular layer until BM and SCM fibers were identified. After, the BM was detached from the anterior border SCM or from the PR when recognized. Below this plane, the antero-medial border of the MPM and PC fat was seen. Blunt dissection was carried out to localize the LN in the compartment between the lateral border of the MPM and the ramus of the mandible. The trajectory and course of the LN were seen at the PR level (along the insertion of the BM and the SCM) (Fig. 2). To carry out measurements of the trajectory and the distance of the LN concerning the PR using radiological studies, three small titanium clips were placed along the LN, and four titanium clips were placed in the most anterior and medial border of the MPM, where it was closer to the junction between the BM and the SMC. The titanium clips at the MPM were placed as follows: Two next to the other, in the upper portion at the level of the lower border of the pterygoid hamulus and two parallel, in the lower portion, at the medial aspect of the ramus of the mandible at the level of the retromolar trigone. (LIGACLIP^®^ Multiple Clip Applier Small 20 clips). All the dissections were performed by Otorhinolaryngologist, head and neck surgeons.

### Radiologic examination

Non-contrast CT of the four specimens was performed on a single-source third-generation CT scanner (SOMATOM X.cite, Siemens Healthcare). The LN was identified where three contiguous titanium clips were placed, laterally. The upper and lower portion of the MPM were identified as two contiguous clips on the supero-anterior border and infero-medial border of the MPM, respectively, as described in the previous section. Multiplanar reconstructions, maximum intensity projection and volume rendering reconstructions were employed for optimal visualization of the relationship of these structures between each other and the adjacent bone. The coronal view was preferred to resemble the surgical position.

**Fig. 3:**
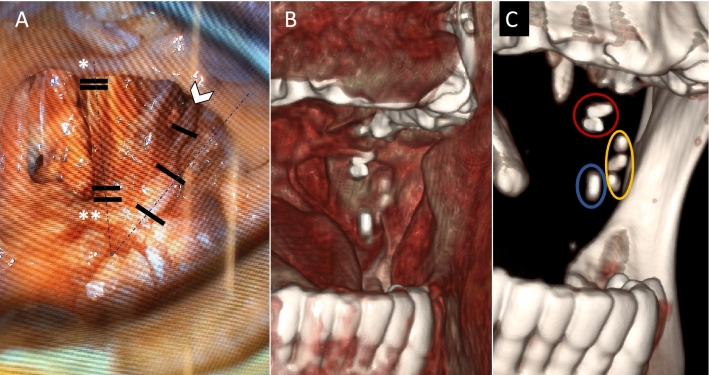
**A** Cadaveric dissection of the left lateral pharyngeal walls shows a diagram of the clips’ position: two contiguous clips were placed at the supero-anterior border of the MPM (*) and two contiguous clips were placed at the infero-medial border of the MPM (**). Three clips were positioned along the LN (White Arrow head). The dot-dash line represents the angle between the trajectory of the LN concerning the MPM. **B**, **C** Cinematic soft tissue (**B**) and bone (**C**) volume rendering CT reconstructions of the same side show the final position of the clips and its relationship to the mandibular ramus (red: superior portion MPM; blue: inferior portion MPM; yellow: lingual nerve)

The distance between the superior clips of the LN and the two superior clips (upper portion of the MPM), and the distance between the inferior clips of the LN and the two clips of the lower MPM portion were measured by the radiologist. The trajectory angle of the LN with respect to the PR was obtained from the intersection between the vector generated between the clips connecting the upper and lower portion of the MPM with the vector generated from the LN clips (Fig. 3).

## Results

A solid tendinous band between de BM and the SCM at the PR level was identified in two out of the eight sides examined (25%). The remaining six sides (75%) exhibited a complete continuity of the BM and the SMC. The LN was found without injuries on all sides. In all the specimens, the LN headed downward, from superior to inferior and from lateral to medial (Fig. 2).

**Fig. 4:**
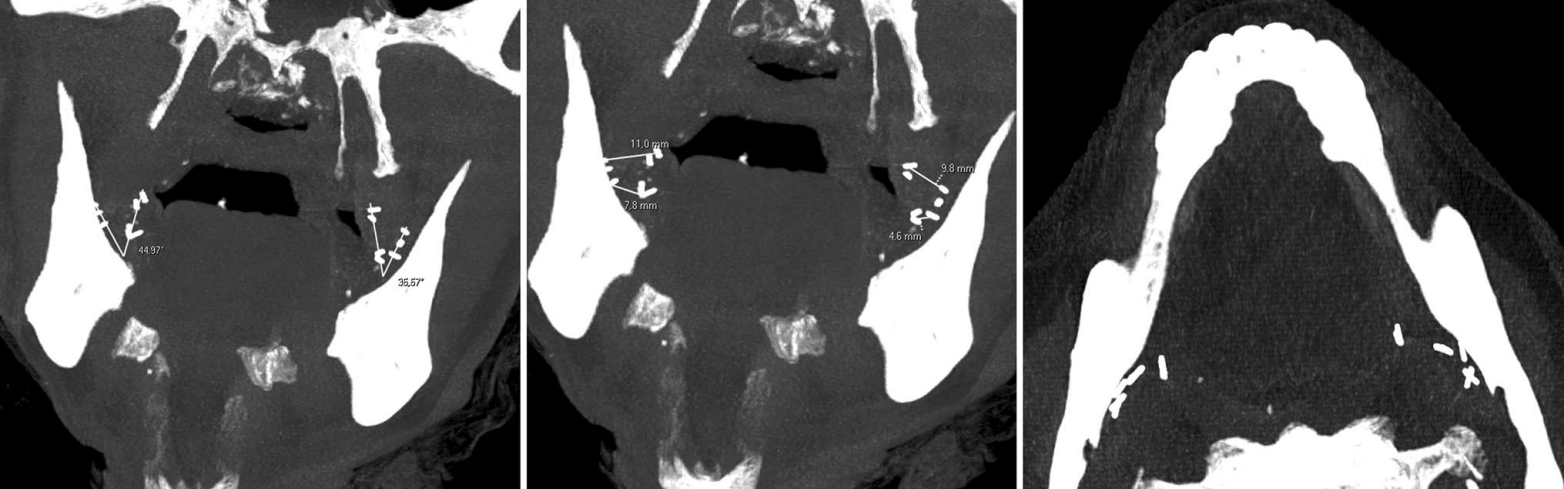
Coronal and axial CT maximum intensity projection reconstruction shows how measurements were performed on a patient

The mean distance from the upper portion of the MPM and superior LN clips was 10.16 ± 2.18 mm (mean ± standard deviation), and the lower area of the MPM to the LN was separated 5.05 ± 1.49 mm (Table 1). The trajectory angle of the LN concerning to the vector that describes the upper portion of the most anterior and medial border of the MPM with its lower part was 43.73º ± 11.29. (Fig. 4).

**Table 1:** Radiological measurements

Patient	Angle LN-PR COR	Distance LN-PR COR (superior portion) (mm)	Distance LN-PR COR (inferior portion) (mm)
1—right	44.97	11	7.8
1—left	35.57	9.8	4.6
2—right	42.89	9.7	3.9
2—left	61.74	14.7	3.8
3—right	28.16	6.5	3.5
3—left	43.38	11.2	4.6
4—right	60.13	9.3	5
4—left	33	9.1	7.2
MEAN	43.73	10.16	5.05
SD	11.29	2.18	1.49

## Discussion

The LN is one of the sensory branches of the mandibular division of the trigeminal nerve. After the mandibular division enters the infratemporal fossa through foramen ovale, it gives off the auriculotemporal, inferior alveolar, and lingual nerves in the infratemporal fossa. The LN receives the chorda tympani approximately 1 cm below the bifurcation of the lingual and inferior alveolar nerves, while carrying taste sensation to the anterior two-thirds of the tongue and parasympathetic innervation to the submandibular and sublingual salivary glands [19]. Although the LN pathway is well-known, its injury during oral surgical procedures and head and neck surgeries are not uncommon, especially in the lingual side of the mandibule [20, 21]. Described as a potential complication during palatal surgery when using the PR as a fixation point [12], in this work, we have sought to explain the anatomy of the LN concerning the oropharyngeal walls and the PR.

In this study, the presence of a tendinous band compatible with the PR was only present in 25% of specimens. The concept of the PR as a tendinous band is not supported by cadaveric studies [22]. Shimada et al. in an anatomical study including 110 sides, concluded that the PR was only partially present in the 28% of specimens. In the rest of the specimens, the PR was absent (36%), or both muscles (BM and SCM) were only separated by fascia (36%). The authors concluded that the mucosa fold on those specimens was redundant mucosa that was accentuated into a vertical fold when opening the mouth [15]. Regarding radiological studies, Brown et al [23] where able to identify a tendinous structure in an axial nasopharyngeal plane compatible with the PR in 63% of the subjects included in their study. They also concluded that MAD treatment response and the amount of maximum advancement improved in subjects without PR. It is essential to bear in mind when performing intra pharyngeal surgery that the PR is an anatomical structure that is only present in approximately one-third of patients, especially if it is to be considered as an anchor point. Nowadays, the best option to study the presence of the PR is through MRI. However, the techniques described are limited to locating its position by identifying the junction of the SCM and the BM, and its prevalence through the imaging studies described differs from anatomical studies [22–24]. For this reason it is necessary to rely on certain bony grips such as the pterygoid hamulus and not refer to the PR.

As described in previous anatomical studies [19–21] and also from a surgical perspective (transoral approach), in all the specimens, the LN passed downward, superiorly–inferiorly and laterally–medially, between the ramus of the mandible and the MPM. All the specimen evaluated showed a well-developed MPM with a typical penniform structure made up of alternating muscular/ aponeurotic layers and tendinous intramuscular sheets as described in an anatomical study performed by El Haddioui et al. [17]. After having crossed through the muscle, the LN takes an antero-medial direction at the anterior border of the MPM and the posterior attachment of the mylohyoid muscle, and most of the time medial, below and behind the third molar, it re-emerges into the mouth lateral to the styloglossus muscle and towards the lateral surface of the tongue, by passing beneath the lower border of the SCM.

Because the PR was not present in all the specimens, and that in those cases in which it was identified, its anatomical position was modified due to a disruption of the buccopharyngeal fascia plane and its loss of tension during dissection, this structure could not be used as a reference measure in the study. The antero-medial border of the MPM was present in all the specimens and its position remained the same after the dissection. Previous anatomic and radiological studies described that the antero-medial border of the MPM is in contact with the antero-lateral border of the SCM [22, 25, 26]. In the specimens included in the study, it was also possible to appreciate the proximity of the antero-medial border of the MPM to the point of intersection between the SCM and the BM.

The relationship of the LN with other structures such as the submandibular duct, the hypoglossal nerve, the inferior alveolar nerve, or the region of mandibular third molar have been described [21, 27, 28]. However, we have not been able to find in the literature studies with similar measurements. Although it had been previously described that there is a potential risk of damaging the LN during palate surgeries, due to its proximity to the lower edge of the PR, the results obtained describe from an objective point of view the anatomic relationship between both structures. The main limitations of this study are the small sample of specimens, the disturbance of the native anatomic location of the structures during dissection, and that all cadaveric specimens were at least 50 years of age, therefore, younger specimens might offer slightly different quantitative results. Although the exact distance between the PR and the LN was not assessed, based on the anatomical findings and previous studies we consider that the distance between the anterior border of the MPM and LN approximates the distance between the PR and the LN.

## Conclusions

When using the PR as anchor point in palate surgeries, the surgeon should keep in mind that the LN runs lateral to it, from superiorly–inferiorly and laterally–medially. It is located at a distance of 10 mm from the superior border of the PR, and that as the nerve descends with an angle of 44º (mean), it progressively approaches the PR, until it is at a distance of approximately 5 mm at the level of the retromolar trigone. ENT surgeons should be familiarized with the palatal anatomy and its most common anatomical landmarks, particularly the LN course to avoid surgical complications while operating at the level of lateral pharyngeal wall.
